# *Porphyromonas gingivalis* Participates in Pathogenesis of Human Abdominal Aortic Aneurysm by Neutrophil Activation. Proof of Concept in Rats

**DOI:** 10.1371/journal.pone.0018679

**Published:** 2011-04-13

**Authors:** Sandrine Delbosc, Jean-Marc Alsac, Clement Journe, Liliane Louedec, Yves Castier, Martine Bonnaure-Mallet, Raymond Ruimy, Patrick Rossignol, Philippe Bouchard, Jean-Baptiste Michel, Olivier Meilhac

**Affiliations:** 1 INSERM (Institut National de la Santé et de la Recherche Médicale) U698, Paris, France; 2 Université Denis Diderot, Paris, France; 3 Service de chirurgie thoracique et vasculaire, Hôpital Xavier Bichat-Claude Bernard, APHP (Assistance Publique Hôpitaux de Paris), Paris, France; 4 Service de chirurgie cardiovasculaire, Hôpital Européen Georges Pompidou, APHP (Assistance Publique Hôpitaux de Paris), Paris, France; 5 Equipe de Microbiologie, UPRES-EA (Unité Propre de Recherche de l'Enseignement Superieur-Equipe d'Accueil) 1254, Université Européenne de Bretagne, Université de Rennes I, Rennes, France; 6 Service de bactériologie et virologie, Hôpital Xavier Bichat-Claude Bernard, APHP (Assistance Publique Hôpitaux de Paris), Paris, France; 7 CHU (Centre Hospitalier Universitaire) de Nancy, CIC (Centre d'Investigation Clinique); CIC9501; Université Nancy, Faculté de Médecine; Inserm, U961, Vandoeuvre lès Nancy, France; Service de médecine vasculaire et hypertension, Hôpital Européen Georges Pompidou, Paris, France; 8 Département de Parodontologie, Service d'odontologie, Hôpital Garancière Rothschild, APHP (Assistance Publique Hôpitaux de Paris), Paris, France; Leiden University Medical Center, Netherlands

## Abstract

**Background:**

Abdominal Aortic Aneurysms (AAAs) represent a particular form of atherothrombosis where neutrophil proteolytic activity plays a major role. We postulated that neutrophil recruitment and activation participating in AAA growth may originate in part from repeated episodes of periodontal bacteremia.

**Methods and Findings:**

Our results show that neutrophil activation in human AAA was associated with Neutrophil Extracellular Trap (NET) formation in the IntraLuminal Thrombus, leading to the release of cell-free DNA. Human AAA samples were shown to contain bacterial DNA with high frequency (11/16), and in particular that of *Porphyromonas gingivalis* (*Pg*), the most prevalent pathogen involved in chronic periodontitis, a common form of periodontal disease. Both DNA reflecting the presence of NETs and antibodies to *Pg* were found to be increased in plasma of patients with AAA. Using a rat model of AAA, we demonstrated that repeated injection of *Pg* fostered aneurysm development, associated with pathological characteristics similar to those observed in humans, such as the persistence of a neutrophil-rich luminal thrombus, not observed in saline-injected rats in which a healing process was observed.

**Conclusions:**

Thus, the control of periodontal disease may represent a therapeutic target to limit human AAA progression.

## Introduction

Abdominal Aortic Aneurysms (AAAs) may be considered as a particular form of atherothrombosis characterized by high levels of proteolytic activity [1], [2], [3] leading to dilation and eventually to rupture of the aortic wall. AAA progression towards rupture is not linear, but usually presents periods of stability alternating with periods of growth (“staccato” growth) [4], [5]. A multilayered IntraLuminal Thombus (ILT) usually lines the aneurysm and presents an interface with the circulating blood components [6]. This blood-ILT interface generates biological activity linked to activation of platelets and the coagulation cascade [7], red blood cell retention and hemoglobin release[8], and neutrophil accumulation leading to retention and/or release of proteases and oxidative activities [6], [9], [10]. Neutrophil activation leads to the release of markers measurable in plasma of patients with AAA, such as MMP-9, elastase-α1 antitrypsin complexes and myeloperoxidase [11]. Elastase that remains associated with the most luminal layer of the ILT inhibits the colonization of the fibrin network by mesenchymal cells, thus impeding the subsequent healing process [12]. We and others have pointed at potential mediators of neutrophil recruitment such as L and P-selectin [7], [13] and chemoattractants such as RANTES, IL-8 and Leukotriene B4 [10]. Nevertheless, these mediators alone do not appear sufficient to explain the abundance of activated neutrophils within the ILT and the staccato progression observed in human AAA.

Recent epidemiological data indicate that chronic periodontitis, the most common form of periodontal disease, is associated with occlusive atherothrombotic plaque progression [14], [15], [16], [17]. However, epidemiological studies linking AAA progression to periodontal disease, or to other sources of weak pathogens, are lacking. Nevertheless, bacterial DNA corresponding to periodontal pathogens has been detected in cardiovascular tissues, including AAA tissue samples [18], [19], [20]. Recently, Aoyama et al. [21] reported that challenge with *P. gingivalis*, but not with *A. actinomycetemcomitans* (an aggressive periodontal pathogen), could promote progression of aortic diameter in an experimental model AAA. There are rational arguments suggesting that weak pathogens, such as periodontal pathogens, could participate in AAA progression:

- As well established in arthropods, coagulation and innate immunity are also interdependent in mammals. Neutrophils and the coagulation system were recently reported to interact and promote bacterial adhesion. This biological process prevents bacterial dissemination and facilitates neutrophil-induced bacterial destruction via the formation of Neutrophil Extracellular Traps (NETs).
- In humans, fibrin and hemoglobin are the most abundant proteins in the ILT. The fibrin network may represent a platform for bacterial adhesion [22], [23] and is a substrate for gingipains [24]. Hemoglobin may also promote the binding of *P. gingivalis* [25] to the ILT and serve as a necessary source of nutriment for various periodontal pathogens, including *P. gingivalis* [26].
- AAA growth is discontinuous [4], suggesting weak transitory but repeated episodes of acute enrichment of the luminal layer of the ILT in biological activity associated with neutrophil accumulation, as suggested by its multilayered aspect. Repeated episodes of bacteremia could be one explanation for this observation.

In the present study, we hypothesized that repeated retention of *P. gingivalis* by the ILT of AAA could enhance neutrophil recruitment and subsequent activation, and thus participate in aneurysmal progression. We first assessed neutrophil activation and NET formation associated with the presence of *P. gingivalis* in human AAA samples. In a second part, we provide an experimental proof of concept, showing that repeated intravenous injection of *P. gingivalis* in a rat model of AAA led to enhanced aortic dilation associated with neutrophil retention and persistence of a non-healing luminal thrombus, mimicking human physiopathology.

## Materials and Methods

### Human tissue and plasma samples

AAA tissues (n = 16) were obtained from patients undergoing surgery and enrolled in the RESAA protocol (REflet Sanguin de l'évolutivité des Anévrysmes de l'Aorte abdominale). All patients gave informed written consent, and the protocol was approved by a French ethics committee (Comité Consultatif de Protection des Personnes dans la Recherche Biomédicale, CCPRB Paris-Cochin, approval no 2095). Control aortas (n = 10) were sampled from dead organ donors with the authorization of the French Biomedicine Agency (PFS 09-007). These control aortic samples were macroscopically normal, devoid of early atheromatous lesions.

Plasma samples of AAA patients were obtained from AMETHYST (Aneurysm Metalloproteinases and Hypertension) study (**Table 1**). Amethyst is an ongoing study promoted by INSERM (Institut National de la Santé et de la Recherche Médicale) that involves a cohort of patients with asymptomatic AAAs (group 1 (n = 11): aortic diameter 3-5 cm; group 2 (n = 21): aortic diameter >5 cm). These patients were age- and sex-matched with healthy volunteers (n = 15). All study participants gave informed consent. The study was approved by an ethic committee (Comité Consultatif de Protection des Personnes dans la Recherche Biomédicale, CCPRB Paris-Cochin approval nos. 1930 and 1931). Exclusion criteria for patients were cancer, infection and any immune-mediated disease. Peripheral blood was drawn in standardized conditions (fasting subjects at rest for 10 minutes, between 8 and 10 AM), with minimal stasis, into prechilled EDTA tubes. No later than 30 minutes after collection, two centrifugations were performed to separate the plasma from the blood cells (2500 rpm, 15 min, 12°C; 2500 rpm, 15 min, 4°C). Plasma samples were stored at −80°C until use.

**Table 1:** Patients' baseline characteristics.

	AAA>5 cm n = 21	AAA<5 cm n = 11	All patients n = 32	p*
Age (years)	76±7	70±9	74±8	0.13
Gender male	19 (90)	11 (100)	30 (94)	0.53
BMI (kg/m^2^)	27.0±3.6	26.7±2.2	26.9±3.2	0.91
Previous history of:				
Diabetes	2 (10)	3 (27)	5 (16)	0.31
Hypertension	15 (71)	8 (73)	23 (72)	1.00
Past or current smoking	18 (86)	10 (91)	28 (88)	1.00
Dyslipidemia	19 (90)	9 (82)	28 (88)	0.59
Concomitant drugs:				
Statins	16 (76)	8 (73)	24 (75)	1.00
Anticoagulants or antiplatelets	5 (24)	0 (-)	5 (16)	0.14
ACEI or ARB	11 (52)	6 (55)	17 (53)	1.00
AAA characteristics:				
Greater diameter (cm)	5.4±0.7	4.3±1.0	5.0±1.0	0.008
Total volume (ml)	141±42	93±37	124±46	0.008
Wall + thrombus volume (ml)	92±41	46±28	76±42	0.003
Intima-media thickness (mm)	0.81±0.20	0.83±0.13	0.82±0.17	0.36

*Values are means ± standard deviation or n (%). AAA: abdominal aortic aneurysm, ACEI: angiotensin converting enzyme inhibitors, ARB: angiotensin II receptor antagonists, wall + thrombus volume: [total - lumen] volumes, p: p-value from the Fisher's (discrete variables) or Mann-Whitney (continuous variables) tests.*

### Determination of human AAA thrombus characteristics and carotid intima-media thickness

AAA diameter and thrombus volume were determined by computed tomographic (CT) angiography using a dedicated software [27]. Briefly, the main steps consisted of: 1) user identification of AAA lumen entry and exit points located near the celiac trunk and iliac bifurcation, respectively; 2) automatic segmentation of 3D lumen; 3) automatic curved multiplanar reformation computation of lumen path; 4) semi-automated aneurysm wall segmentation on curved multiplanar reformations based on active contour processing; 5) interactive contour validation and editing if needed. Finally, 3D mathematical models of the AAA components were reconstructed and automatic calculation of maximal AAA diameter, and thrombus volume were performed. All CT examinations were anonymized and processed by an experimented CT technologist blinded to the radiology report. Repeated measurements allowed calculation of coefficients of variation (CV) for AAA size (16%, intra-class coefficient of correlation: 0.88) and thrombus size (10%, intra-class coefficient of correlation: 0.85).

Carotid intima media thickness (IMT): ultrasonography of both left and right common carotid arteries was performed using a high-resolution B-mode system with a 7.5-MHz linear array transducer (ATL Apogee 800+). The arterial wall segments were assessed in a longitudinal view, strictly perpendicular to the ultrasound beam, with both walls clearly visible to achieve diameter measurements. The actual IMT measurements were performed on the far wall along a minimum 10-mm length of an arterial segment with a high-quality image automatic acquisition using IOTEC software (IODP). Adventitia-to-adventitia diameter and intraluminal diameter of the common carotid artery were also measured. The intra-observer reproducibilities were 3.6±4%.

### Human aneurysmal conditioned medium

Briefly, the ILT was dissected into three parts [28]: luminal (at the interface with the circulating blood), intermediate, and abluminal layers. The media of AAA and control aortas was separated from the adventitia and each layer was cut into small pieces (5 mm^3^), separately incubated (24 hours at 37°C) in a standardized volume (6 mL/g of wet tissue) of RPMI 1640 medium supplemented with antibiotics and an antimycotic. The conditioned medium was centrifuged, and the supernatant was aliquoted and frozen at −80°C until use.

### *Porphyromonas gingivalis* culture

*P. gingivalis* strain (T103683) was purchased from the Collection de l'Institut Pasteur (Paris, France) and was grown on M20 medium, consisting of 3% (w/v) tryptone, 1.5% (w/v) agar, 2% (w/v) yeast extract, 0.05% (w/v) cysteine hydrochloride, 05% (w/v) glucose and 2.5% (v/v) hemin solution (0.1% (w/v) hemin chloride, 4% (v/v) triethanolamine), in an anaerobic chamber at 37°C (Bio-Merieux, Lyon, France). Bacteria were subcultured once a week, 1 mL of the cellular suspension was centrifuged (5,000 g, 5 minutes) and resuspended in 12 mL of M20 medium.

### Experimental model of AAA

Experimental AAA was induced by implanting a segment of sodium dodecyl sulfate (SDS)-decellularized guinea pig aorta in rat aorta as previously described [29]. Briefly, guinea pig infrarenal aortas (1.5 cm) were dissected out under deep pentobarbital anesthesia and decellularized by SDS treatment (0.1%, overnight 4°C). The next day after washing in saline, decellularized guinea pig aortas were orthotopically transplanted into the Lewis rat. One week after the surgery, *P. gingivalis* (10^7^ CFU in 500 µL of saline) suspension or saline alone was injected once a week via the jugular vein for 4 weeks. Two days after the fourth injection, rats were anaesthetized by sodium pentobarbital (50 mg/kg, IP) and sacrificed. Blood was collected in citrated tubes and the diameters of the AAA and the thoracic aorta were measured before removal. The aneurysmal wall was fixed in paraformaldehyde (3.7%) for immunohistochemical analysis or incubated for 16 hours in RPMI-1640 at 37°C (Invitrogen, Cergy-Pontoise, France) (6 mL/g of wet tissue), in order to obtain conditioned medium. The conditioned medium was centrifuged at 3,000 g for 10 minutes at 20°C and the supernatant was then aliquoted and stored at −80°C until use.

### Animal sample size calculation

The study was designed with 80% power to detect a relative 50% difference in aneurysmal size between *P. gingivalis* and saline groups. Statistical testing was performed at the two-tailed (alpha) level of 0.05 using a t-test. Based on preliminary data indicating that the average aneurysmal size at 5 weeks after xenograft surgery was 5.41 mm, standard deviation: 1.83, we used 11 rats for each group (*P. gingivalis* or saline). A computer-based randomization was used to allocate P. gingivalis or saline injection to each rat.

### Immunofluorescence

Human AAA, normal aorta and rat aorta were fixed in paraformaldehyde (PFA) 3.7%, embedded in paraffin and sectioned at 6 µm. The sections were deparaffinized in toluene and hydrated in graded series of ethanol. After blocking in 5% goat serum, mouse anti-human elastase clone 265-3K1 (1 µg/ml, Hycult Biotechnology), rabbit anti-MPO (1∶100, Dako), mouse anti-Histone H1 (4 µg/ml, Santa-Cruz Biotechnology), rabbit anti-Histone H4 Cit3 (1∶200, Millipore) or rabbit anti-*P. gingivalis* (1∶100, a generous gift from Dr Bonnaure-Mallet) were applied to sections and incubated for 1 hour at room temperature. After washing with PBS, appropriate secondary antibodies (goat anti-mouse or anti-rabbit conjugated with either Alexa 555 or 488, 2 µg/ml) were incubated for 1 hour at room temperature. DAPI (100 ng/mL) was added for 15 minutes and slides were mounted using Fluoprep mounting medium (Dako). All steps are separated by 3 washes by PBS. The method of terminal dUTP nick-end labeling (TUNEL) was used to visualize DNA fragmentation (Roche Diagnostic, Meylan, France).

### Western-Blot

Twenty µg of proteins of human tissue-conditioned medium (thrombi and arterial wall of AAA and normal aortas) were loaded on a 10% polyacrylamide gel for their separation under denaturing (SDS) and reducing conditions, before been transferred to a nitrocellulose membrane (Hybond, Amersham Biosciences, England). After blocking in 5% nonfat dried milk, the membrane was incubated with rabbit polyclonal anti- citrullinated histone H4: (1∶1000, Millipore, France) for 1h30, and then with goat anti-rabbit secondary antibody conjugated to horseradish (1/20000; Jackson ImmunoResearch Laboratories, England) for 1 hour at room temperature. Detection was performed by using ECL reagents (Amersham Biosciences, England).

### Determination of cell-free DNA concentrations

Cell-free DNA (cf-DNA) concentration was determined in the conditioned medium of aortic samples and in plasma, in both humans and in the experimental AAA model in rats, using Quant-it™ Picogreen® ds DNA Reagent (Invitrogen). Briefly, 10 µL of samples and Lambda DNA standard (1 ng/mL - 1 µg/mL) were diluted in TE buffer (200 mM Tris-HCl, 20 mM EDTA, pH 7.5, 100 µL final) before addition of 100 µL Picogreen® dsDNA reagent. After mixing, and incubation for 5 minutes at room temperature in the dark, the fluorescence was measured using a microplate reader (excitation 480 nm, emission 520 nm). Intra-assay coefficient of variation (CV) was estimated at 3.8%.

### Determination of Myeloperoxidase (MPO), MMP-9 and MPO-DNA complexes

The concentration of MPO in conditioned medium and in plasma of rats was determined using the rat MPO ELISA kit from Hycult Biotechnology (Uden, The Netherlands, intra-assay CV: 3.66%). MMP-9 activity in conditioned medium and plasma of rats was determined by gelatin zymography [30]. Briefly, 20 µL of samples were loaded onto an SDS-10% polyacrylamide gel containing 1% of type 1 gelatin. After electrophoresis, SDS was eliminated by a 2.5% triton X-100 solution (2x 30 min). The gels were rinsed with H_2_O and then incubated for 20 hours in a buffer containing 50 mM Tris and 2.5 mM CaCl_2_ before staining by Coomassie blue.

MPO-DNA complexes were quantified in human and rat samples (conditioned medium and plasma) by combining two different ELISA tests as previously described [31]. First, MPO was captured onto a 96-well plate coated with a monoclonal antibody against MPO (rat or human MPO ELISA kit, Hycult Biotechnology). Diluted samples (1∶10) were added to the plate and incubated for 1 hour at room temperature, and non captured material was eliminated by thorough washing steps. Second, a peroxidase-labeled anti-DNA monoclonal antibody (component number 2 of the Cell death detection ELISA kit, Roche) was added and incubated for 2 hours with gentle shaking. After washing, the peroxidase substrate ABTS (Sigma-Aldrich) was added, and the absorbance at 405 nm was measured after 30 minutes of incubation in the dark. Intra-assay CV was estimated at 4.22%.

In order to determine the percentage of cf-DNA deriving from NETs, cf-DNA concentration was evaluated in the samples before and after MPO immunocapture.

### Determination of bacterial endotoxin

Endotoxins released by the ILT and the residual arterial wall into the conditioned medium (n = 16) were quantified using the Limulus Amebocyte Lysate (LAL) chromogenic endpoint assay (Hycult Biotechnology) according to the supplier's instructions. Briefly, samples diluted at 1∶5 (50 µL final) were incubated with LAL reagent (50 µL) for 30 minutes at room temperature and a stop solution was added before reading on a spectrophotometer at 405 nm.

### DNA extraction and bacterial DNA amplification by PCR

Samples of AAA ILT, media and adventitia and control aortas were pulverized using a freezer mill (Spex Certiprep Ltd) and DNA was extracted from the powder by the QIamp DNA blood Midi kit (Qiagen). Briefly, 100 mg of tissue were incubated with lysozyme (20 mg/mL) diluted in 20 mM Tris-HCl pH 8.0, 2 mM EDTA, 1.2% Triton for 30 min at 37°C. Samples were then incubated with 20 µL of proteinase K) (Qiagen) at 56°C until the tissue was completely digested and the protocol for DNA extraction was then followed according to the manufacturer's instructions. Ten ng of DNA were loaded on a 1% agarose gel and stained by ethydium bromide for quality control before amplification. The same protocol was used to isolate DNA of rat aortas.

The extracted DNA was amplified using either an ubiquitous primer set that matches almost all bacterial 16S ribosomal RNA (*Forward*: 5′-AGC GAT GGT AGC AAT ACC TGT C-3′; *Reverse*: 5′-TTC GCC GGG TTA TCC CTC-3′, Tm 55°C) or by a pair of specific primers corresponding to a sequence encoding 16S rRNA of *P. gingivalis* (*Forward*: 5′-AGG CAG CTT GCC ATA CTG CG-3′; *Reverse*: 5′-ACT GTT AGC AAC TAC CGA TGT-3′, Tm 60°C) [32]. Briefly, PCR was carried out in a mixture containing 7 µL of DNA (50 ng), 7 µL of H_2_O, 4 µL of Master Mix and 2 µL of 0.2 mM PCR primer set and amplification (50 cycles) was carried out by real time PCR using a LightCycler® system with SYBR green detection (Roche Applied Biosystems).

Products of amplification were then analyzed by electrophoresis on a 1% agarose gel stained by ethydium bromide. Genomic DNA extracted from *P gingivalis* strain 381was used as a positive control.

### Determination of anti- *P. gingivalis* antibodies by ELISA

The presence of antibodies against *P. gingivalis* in conditioned medium and in serum was investigated as previously described [33]. Briefly, a suspension of *P. gingivalis* bacteria was centrifuged (10,000 g for 30 min at 4°C). The pellet was washed by 0.05 M sodium carbonate buffer and then resuspended in the same buffer to an optical density of 1.0 at 640 nm. The bacteria were heated at 60°C for 45 min, diluted 1∶10 in sodium carbonate buffer and dispensed in 96-well plates. The plates were then incubated at 37°C for 4 hours and then overnight at 4°C. The excess bacteria were removed by washing in 0.005% Tween 20 in PBS and plates were allowed to air-dry before storage at−20°C until use. Each serum sample (dilution 1∶100, in 1% BSA-PBS) was added to the *P. gingivalis*-coated plate and incubated at 37°C for 2 hours. After washing, 100 µL of peroxidase-labelled rabbit anti-human IgG,A,M (dilution 1∶500 in 1% BSA-PBS) were added to each well and incubated for 2 hours at 37°C. After washing, TMB was used as substrate for peroxidase and the reaction was stopped with 0.5N H_2_SO_4_ before reading at 450 nm. Intra-assay CV was estimated at 9.32%.

### Neutrophil isolation and *in vitro* stimulation of NETs formation

Human neutrophils were isolated from healthy donors using a dextran/Ficoll method. Briefly, leukocytes were separated from red blood cells by sedimentation after hemaglutination in 1% dextran (20 minutes, room temperature) followed by a Ficoll-Paque centrifugation (616 g, 25 minutes, 20°C) and hypo-osmotic lysis of erythrocytes [34]. After washing in PBS, cells were counted on a Hemalog H1 device (Technicon Instruments Corp., Tarritown, NJ, USA) and adjusted to 1.10^6^ cells/mL in HBSS without Ca2^+^. Cells were seeded in 8-well Lab-tek® chamber slides (Permanox, Thermo scientific) at 250,000 cells/well for immunofluorescence staining and in a 96-well plate for cell-free DNA determination. After adhesion (1 hour, 37°C), neutrophils were activated by adding formyl-Methionyl-Leucyl-Phenylalanine (fMLP, 100 nM) or *P. gingivalis* (1.10^5^ to 1.10^7 ^CFU/mL) for 2 hours at 37°C. The conditioned media were centrifuged (2,500 g, 10 minutes, room temperature) and the supernatants were stored at −20°C. Neutrophils were fixed in PFA 3.7% for immunofluorescence.

### Statistical analysis

Results are expressed as box plots in which the boxes represent the 25^th^ and 75^th^ percentiles, the line within the box represents the median value and the lines outside the boxes represent the 5^th^ and the 95^th^ percentiles. Differences between control and AAA subjects or saline- and *P. gingivalis*- injected rats were assessed by the Mann-Whitney non-parametric test (Prism 5, GraphPad software). The correlations were determined by the Least Squares method. Statistical significance was accepted when p<0.05.

## Results

### AAA intraluminal thrombus is enriched with neutrophil extracellular traps

**Figure 1:**
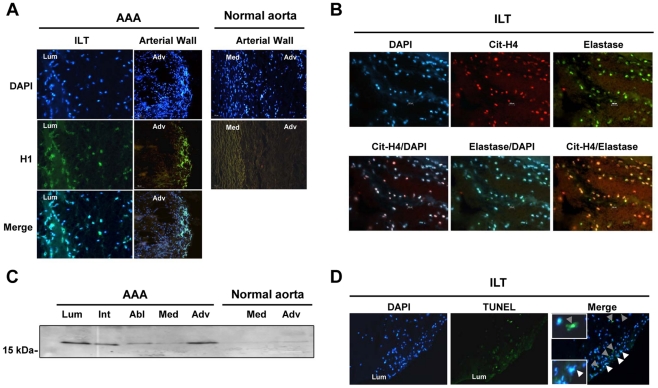
Characterization of neutrophil extracellular traps (NETs) in human AAA samples. The presence of NETs in AAA intral-luminal thrombus (ILT) and in aneurysmal wall was demonstrated by co-immunostaining of histone H1, citrullinated histone H4 (cit-H4) and elastase. Nuclei were stained with DAPI (100 ng/ml, 15 minutes). (A) Immunostaining for histone H1 (green) showing destructured nuclei in the luminal (lum) part of ILT as well as in the adventitia (Adv). (B) Immunostaining for cit-H4 and elastase demonstrates specific neutrophil activation prior to DNA expulsion. (C) The release of cit-H4 was analyzed by western blot in the conditioned medium from the different layers of ILT (luminal: Lum, intermediate: Int and abluminal: Abl) and of the remaining aortic wall (media: Med and adventitia: Adv). (D) Terminal-transferase dUTP Nick End Labelling (TUNEL) staining in the luminal part of ILT (green). TUNEL-positive cells are indicated by grey arrowheads. Extracellular nucleosomes (NETs) are indicated by white arrowheads. Merged images were obtained using Archimed software.

Pathogen-induced neutrophil activation was recently reported to induce the formation of NETs [35], consisting of extracellular, highly decondensed chromatin (histones and DNA) associated with neutrophil granule proteins (elastase, myeloperoxidase, etc.) [36], and thought to play a pivotal role in anti-bacterial defense. Immunodetection of histone H1 revealed strong staining in the luminal part of the ILT, associated with disorganized nuclei, stained by DAPI (**Figure 1A**). No staining was observed in the abluminal part of the ILT, almost devoid of cells. In contrast, immunostaining revealed intense histone H1 positivity in the adventitia of AAA relative to that of control aortas. Interestingly, H1 staining was not observed in intact nuclei due to the low accessibility of the histones within condensed chromatin to the antibody, under the experimental conditions used for immunofluorescence staining (performed without permeabilization).

Histone citrullination (H3 and H4) was previously reported to be a hallmark of NET formation, accompanying chromatin decondensation in neutrophils [37]. Citrullinated histone H4 (Cit-H4) immunostaining was observed in areas of neutrophil accumulation, as shown by elastase co-staining, mainly in the luminal part of the ILT (**Figure 1B**) and in the adventitia of AAA (data not shown). The release of citrullinated histones was then assessed by western-blot in the conditioned medium of each layer of the ILT and of the arterial wall (**Figure 1C**). Cit-H3 was shown to be mainly released by the luminal part of the ILT and by the adventitia of AAA samples, whereas conditioned medium from control aortic wall did not contain detectable levels of Cit-histones. Finally, detection of fragmented DNA by TUNEL showed a positive staining in the luminal part of the ILT that did not exactly co-localize with NETs (**Figure 1D**).

### Increased cell free-DNA (cf-DNA) concentration in conditioned medium and in plasma of AAA patients *vs* healthy subjects

**Figure 2:**
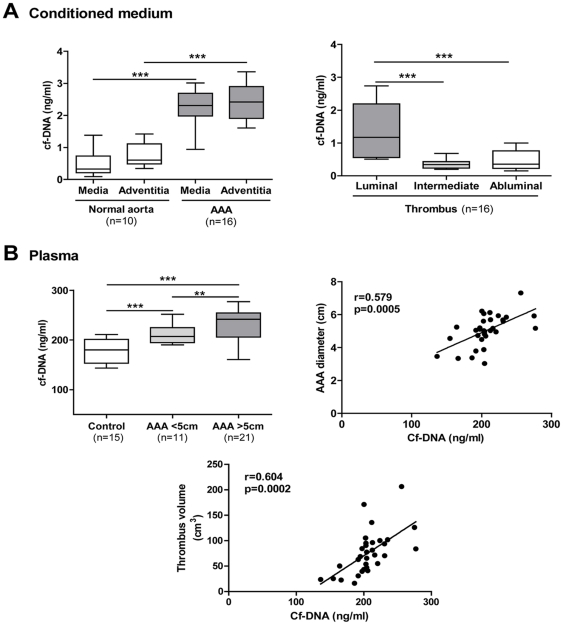
Increased cell-free DNA (cf-DNA) in the conditioned medium and in plasma of human AAA. (A) The concentration of cf-DNA was determined in the conditioned medium obtained from the arterial wall of control and aneurysmal aortas, from AAA thrombus and (B) in plasma of healthy subjects and patients with a small (<5 cm) or a large (>5 cm) AAA. Results are presented as box plots in which the median is shown. **p<0.01; ***p<0.0001 (Mann-Whitney analysis). The correlation (n = 32) between AAA diameter, thrombus volume and cf-DNA concentration were obtained by the Least Squares method.

As shown for citrullinated histone-H3, we postulated that NET formation in the tissue might be reflected by the release of cf-DNA into the conditioned medium [38]. The release of cf-DNA by AAA arterial wall (media and adventitia) was significantly higher relative to that of normal aortas (**Figure 2A**). The luminal part of the ILT was the main source of cf-DNA as compared to intermediate and abluminal layers (p<0.0001). These results are consistent with the enrichment of the luminal layer in neutrophils [12].

In plasma, the concentration of cf-DNA was significantly increased in patients with AAA compared to that of control subjects (p<0.0001), and positively correlated with AAA diameter (n = 32, r = 0.579, p<0.01), and thrombus volume (n = 32, r = 0. 604, p<0.01, **Figure 2B**). A significant difference was observed between large (>5 cm) and small (3–5 cm) AAA (p<0.01).

### Increased circulating MPO-DNA complexes in AAA

**Figure 3:**
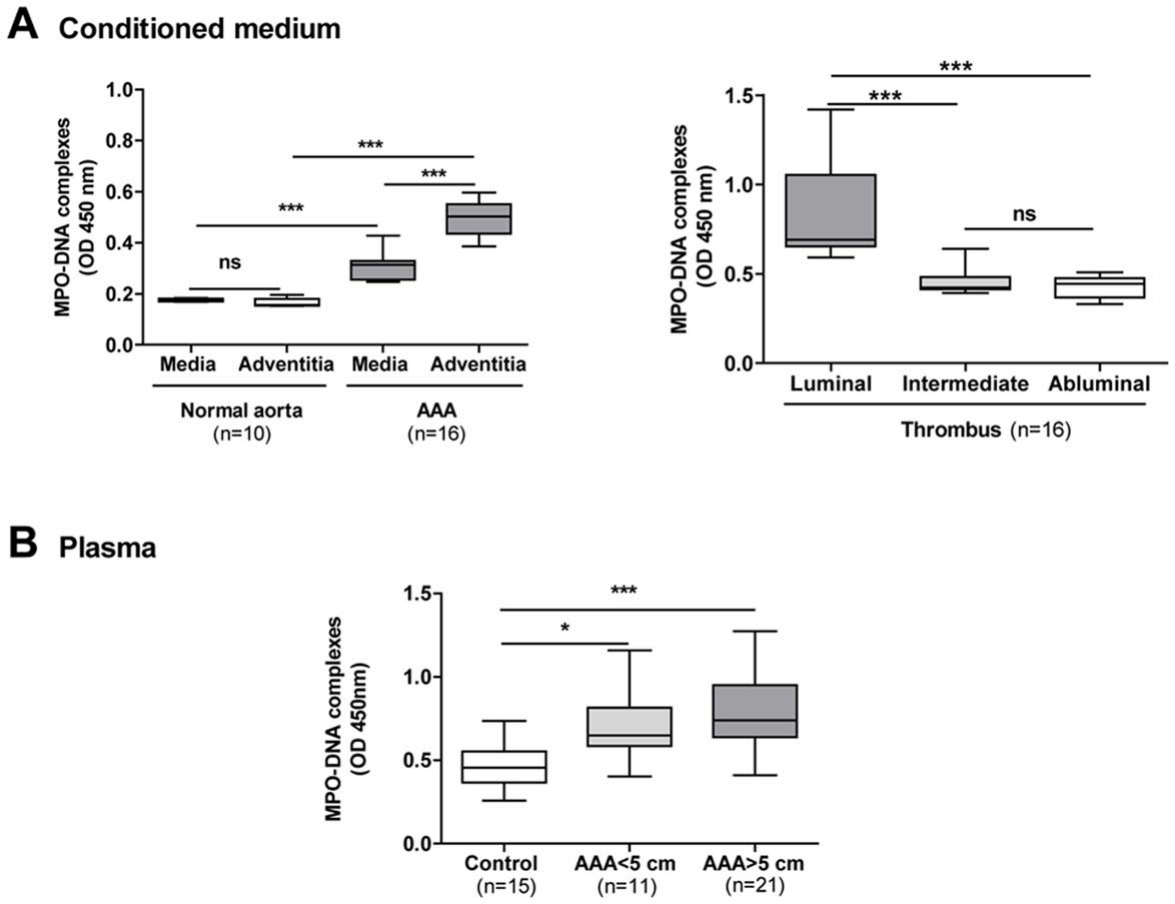
MPO-DNA complexes in conditioned medium and in plasma of human AAA. MPO-DNA complexes released by the intra-luminal thrombus (ILT) of AAA and by the arterial wall of control aorta and AAA were quantified in the conditioned media (A) as well as in plasma (B). A sandwich ELISA was used, consisting of an anti-human MPO for immunocapture and a peroxidase-conjugated anti-DNA antibody for detection. Results are presented as box plots in which the median is shown. *p<0.05, **p<0.01; ***p<0.0001 (Mann-Whitney analysis).

Although often used to quantify circulating NETs [38], cf-DNA assay measures all types of DNA able to interact with Picogreen, whatever its origin (coming from NETosis, necrosis or apoptosis of cell types other than neutrophils). In order to show that cf-DNA did indeed reflect circulating NET content, we measured the MPO-DNA complexes, by immunoprecipitation of MPO followed by detection with an anti-DNA conjugated with horseradish peroxidase [31] in conditioned medium (AAA and normal aorta samples) and in plasma. The concentration of the MPO-DNA complexes was significantly higher in conditioned medium of aneurysmal wall as compared to that of normal aorta (**Figure 3A**), and also in that from the luminal thrombus layer compared to that from intermediate and abluminal layers.

In plasma, the concentration of MPO-DNA complexes was significantly higher in patients with AAA than in controls (**Figure 3B** **):** p<0.05 between controls and small AAA patients, p<0.0001 between controls and large AAA patients. No significant difference between small and large aneurysms was observed. Moreover, the concentration of MPO-DNA complexes was positively correlated with levels of cf-DNA (r = 0.545, p = 0.0015). The concentration of cf-DNA was also determined in plasma samples before and after MPO immunocapture, in order to evaluate the proportion of cf-DNA originating from NETs. Our results show that approximately 20% of plasma cf-DNA was MPO-associated DNA (data not shown), suggesting that a part of cf-DNA release is dependent on NET formation.

### Presence of bacteria in AAA samples

**Figure 4:**
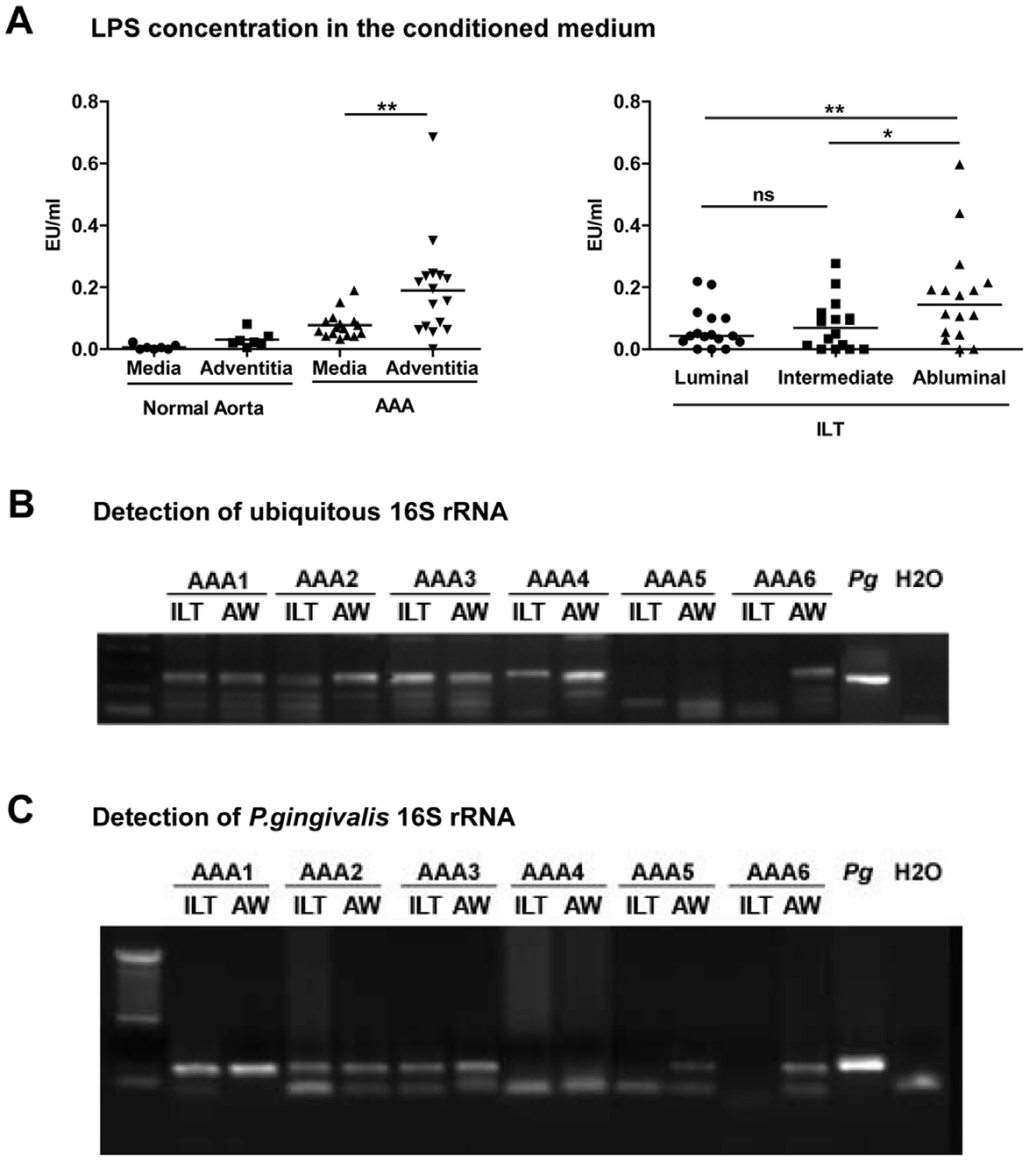
Detection of bacteria in human AAA samples. (A) Endotoxin levels from gram-negative bacteria were quantified in the conditioned medium of the Intra-luminal thrombus (ILT) and the arterial wall of AAA (n = 16) and control aortas (n = 10) using the Limulus Amebocyte Lysate chromogenic assay kit. *p<0.05; **p<0.01 (Mann-Whitney Analysis). DNA was extracted from the ILT and associated arterial wall before amplification by PCR using a ubiquitous set of primers targeting bacterial 16S rRNA (B) or *Pg* 16S rRNA (C) gene. Amplification products were separated by electrophoresis in a 1% agarose gel.

**Figure 5:**
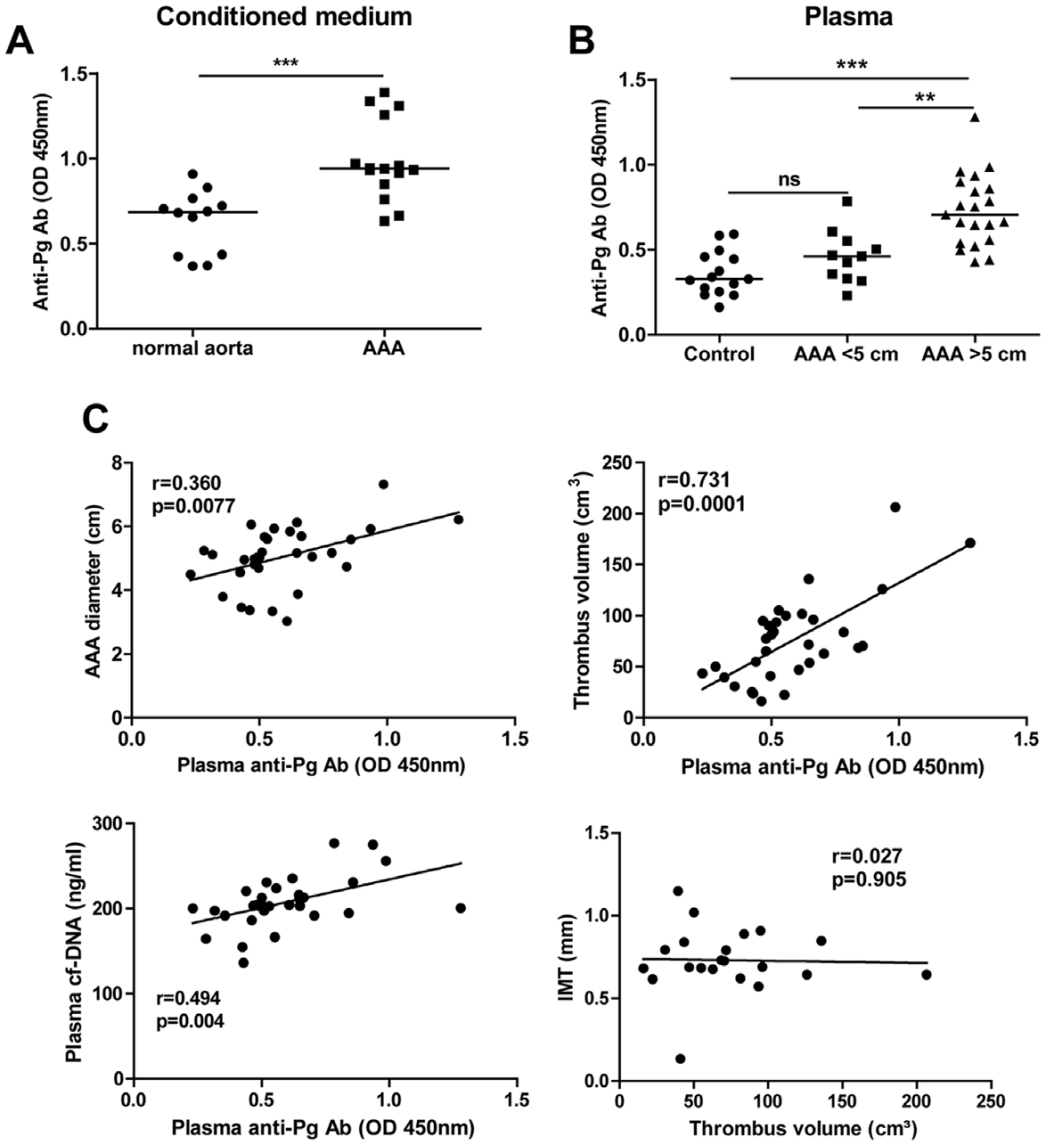
Anti-*P. gingivalis* antibodies (anti-*Pg* Ab) in human AAA. The presence of antibodies against *Pg* was investigated by a custom ELISA in the conditioned medium of adventitia (A) and in serum of patients with AAA or controls (B). **p<0.01; ***p<0.0001 (Mann-Whitney analysis). The correlations (n = 32) between AAA diameter, thrombus volume, plasma cf-DNA and anti-*Pg* Ab or Intima Media Thickness (IMT) and thrombus volume (C), were determined by the Least Squares method.

Bacteria represent one of the major triggers of NET formation. To test our hypothesis that AAA ILT could be a substrate for bacterial retention and therefore participate in the aneurysmal progression, we first quantified endotoxin (lipopolysaccharide or LPS) in the conditioned medium of the different layers of the ILT (**Figure 4A**) by the Limulus Amebocyte Lysate assay. Out of 16 AAA ILT samples, only 4 of them had undetectable levels of bacterial LPS (**Figure 4A**). The abluminal thrombus layer was shown to release more LPS than its luminal layer (p = 0.005). The presence of bacteria was then investigated by PCR on the total DNA extracted from the ILT and arterial wall samples (in both control and aneurysmal aortas). Amplification of DNA encoding for bacterial 16S ribosomal RNA was performed using specific primers. 10/16 thrombi and 11/16 aneurysmal walls were positive for bacterial DNA (**Figure 4B**). *P. gingivalis* is the major anaerobic pathogen responsible for periodontal disease and may produce chronic bacteremia subsequent to chewing or toothbrushing. *P. gingivalis* was detected by PCR in 6/16 thrombi and 7/16 aneurysmal wall tested (**Figure 4C**). In contrast, all control aortic walls were negative for 16S ribosomal RNA for all bacteria and *P.gingivalis* in particular (data not shown). In addition, *P. gingivalis* was indirectly assessed by quantification of antibodies immunoreactive against *P. gingivalis* either released from AAA adventitia (containing tertiary lymphoid organs or ATLOs) [2] or contained in the serum of patients with AAA versus controls. **Figure 5A** indicates that adventitia from aneurysmal aortas released higher amounts of anti-*P. gingivalis* immunoglobulins relative to adventitia from normal aorta. Furthermore, serum of AAA patients contained more immunoreactive antibodies against *P. gingivalis* compared to control subjects (**Figure 5B**). Although analysis of the correlation between adventitial and serum antibodies against *P. gingivalis* could only be performed on a limited number of samples (n = 15), a positive association was found (r = 0.54, p = 0.039). Strong positive correlations were observed between the titer of anti-*P. gingivalis* immunoglobulins in serum and cf-DNA, AAA diameter and thrombus volume (**Figure 5C**, n = 32). It is noteworthy that no correlation (**Figure 5C**) between the thrombus volume and the intima-media thickness was observed (r = 0.027, p = 0.905).

### *P. gingivalis* promotes NET formation *in vitro*

**Figure 6:**
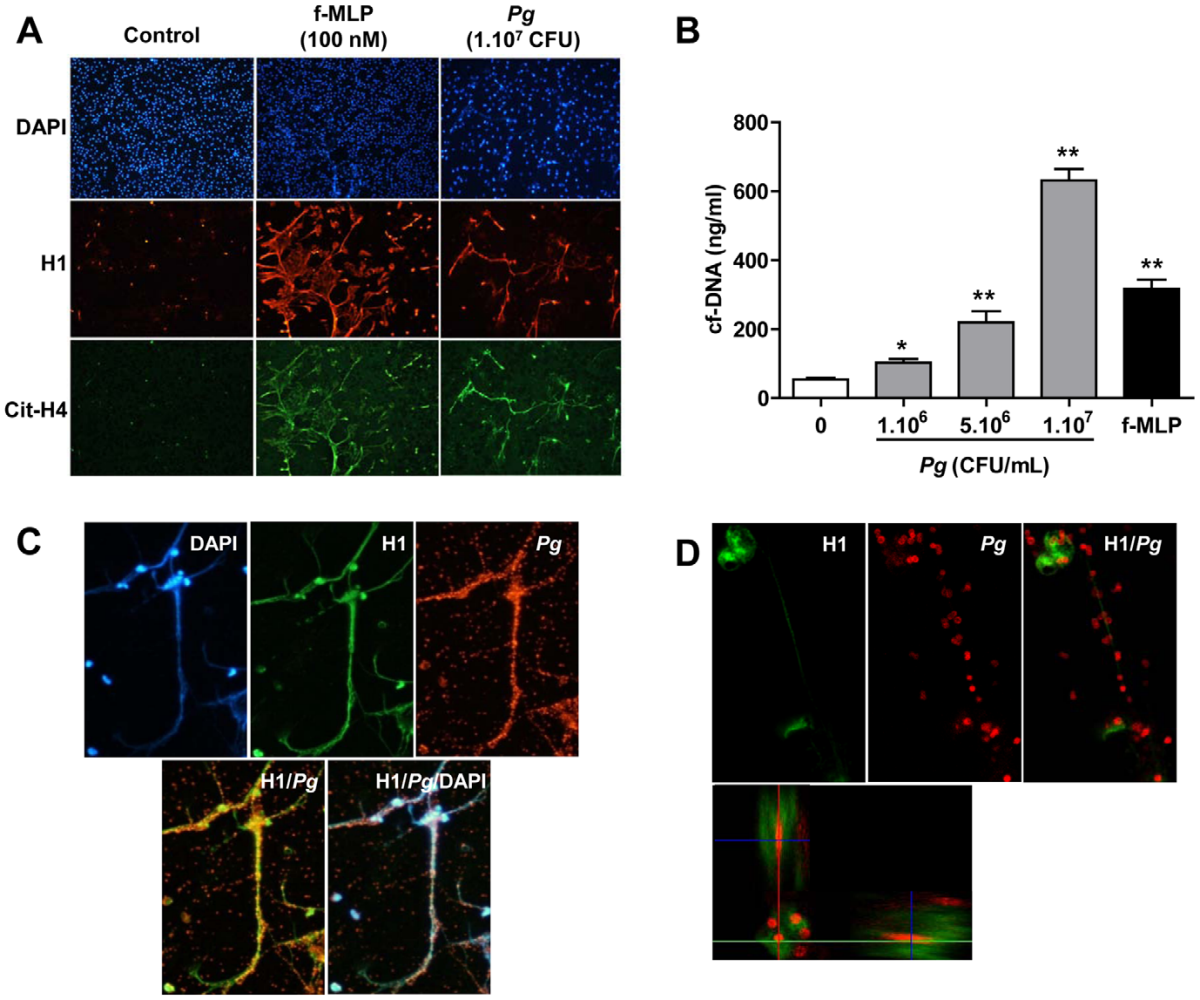
*P. gingivalis* (*Pg*) promotes NET formation. Freshly isolated human neutrophils were plated on Lab-tek® chamber slides and then stimulated or not by f-MLP (100 nM), a bacterial peptide used as a positive control, or by *Pg* (1.10^7^ CFU) for 2 hours at 37°C. (A) Immunofluorescence detection of histone H1 (red) and citrullinated histone H4 (green) was performed without permeabilization. (B) Cell-free DNA (cf-DNA) concentration was determined in the culture medium of neutrophils stimulated or not either with f-MLP (100 nM) or increasing concentrations of Pg. *p<0.05; **p<0.01 *vs* ctl (Mann-Whitney analysis). The trapping of Pg (red) by externalized nucleosomes (histone H1, green) was visualized by epifluorescence (C) and confocal microscopy (D). The bottom panel represents a virtual section constructed according to the Z axis, confirming the intracellular presence of *Pg* subsequent to phagocytosis by neutrophils.

The presence of *P. gingivalis* in AAA samples shown by PCR and the correlations between anti- *P. gingivalis*/cf-DNA and anti- *P. gingivalis*/AAA diameter led us to hypothesize that these bacteria could participate in the chronic renewal of the ILT *via* recruitment and activation of neutrophils. We found that *P. gingivalis* were able to trigger the formation of NETs, as shown by immunofluorescent staining of histone H1 and citrullinated histone H4 (**Figure 6A**). *P. gingivalis* promoted neutrophil DNA expulsion in a dose-dependent manner as shown by quantification of cf-DNA in the supernatant of neutrophils incubated with different concentrations of *P. gingivalis* (**Figure 6B**). Induction by *P.gingivalis* of NET formation and subsequent trapping was further demonstrated by epifluorescence (**Figure 6C**) and confocal microscopy (**Figure 6D**).

### Chronic *P. gingivalis*-bacteremia induces neutrophil recruitment in experimental AAA

**Figure 7:**
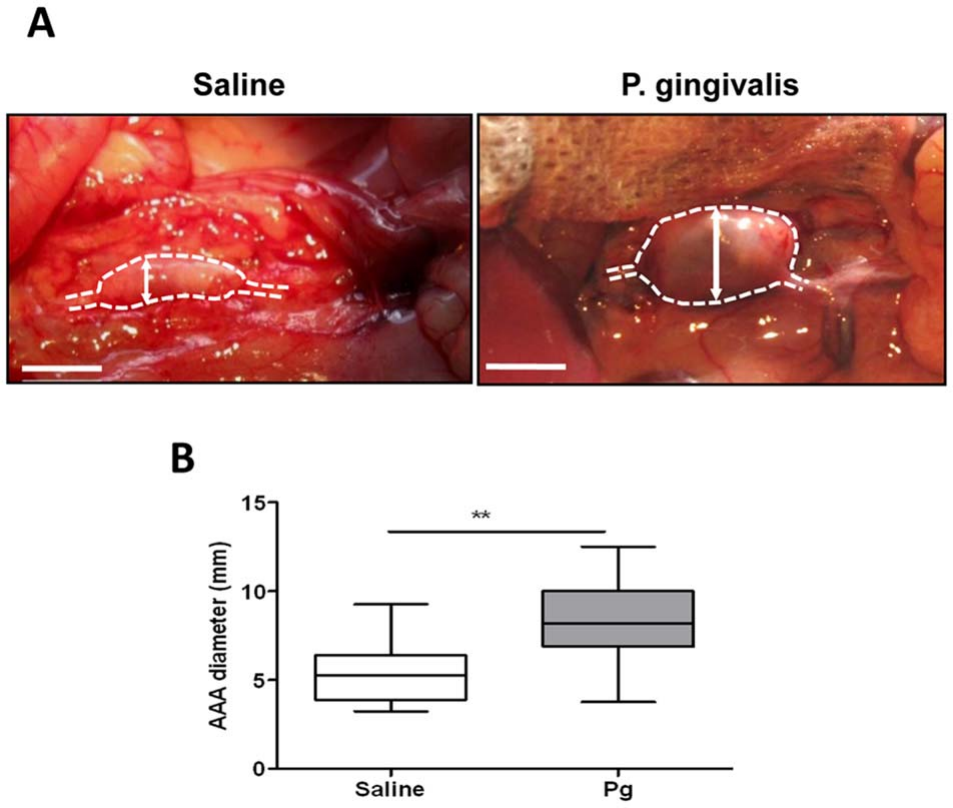
Chronic *P gingivalis* (*Pg*) infection fostered AAA development in rats. (A) Experimental AAA was induced by implanting a segment of a SDS-decellularized guinea pig aorta in the rat aorta. Rats were or not infected weekly with *Pg* (1.10^7^ CFU/500 µL/rat, for 4 weeks). (B) At the end of treatment, rats were anaesthetized for blood sampling and sacrified after measuring AAA diameter. Results (n = 10) are presented as box plots in which median is shown, **p<0.01 (Mann-Whitney Analysis).

**Figure 8:**
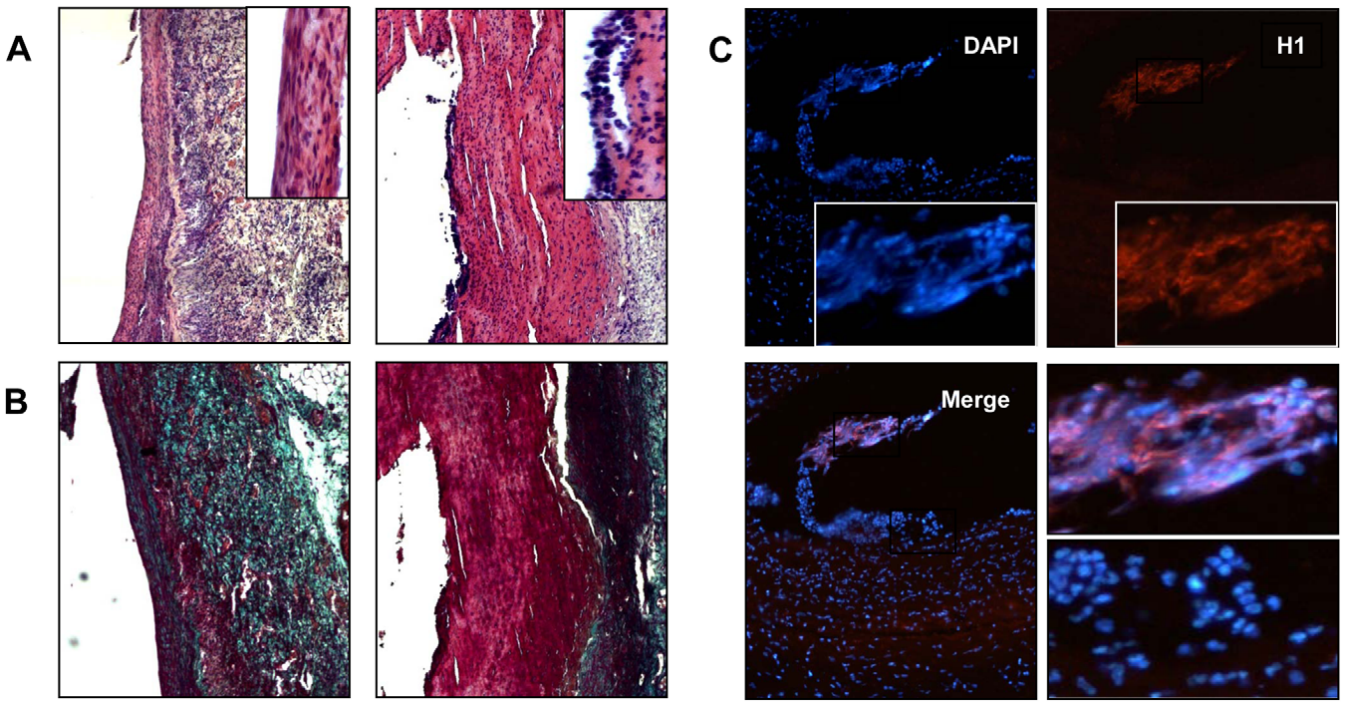
*P. gingivalis* (*Pg*) infection promoted neutrophil recruitment, NET formation and inhibited healing. (A) Hematoxylin/Eosin staining showing the presence of a thrombus and neutrophil accumulation at its luminal pole (right, inset) in *Pg*-infected rats. The presence of mesenchymatous cells is observed in saline-injected rats (left, inset). (B) Masson's trichrome staining. Fibrosis associated with healing is observed in green in saline-injected rats whereas red staining highlights the presence of a thrombus in *Pg*-infected rats. (C) Immunostaining for histone H1 (red), nuclei appear in blue (DAPI). Merged images show the presence of extracellular H1 associated with disorganized DNA (inset), but also intact neutrophils characterized by their multilobed nuclei (bottom, right).

**Figure 9:**
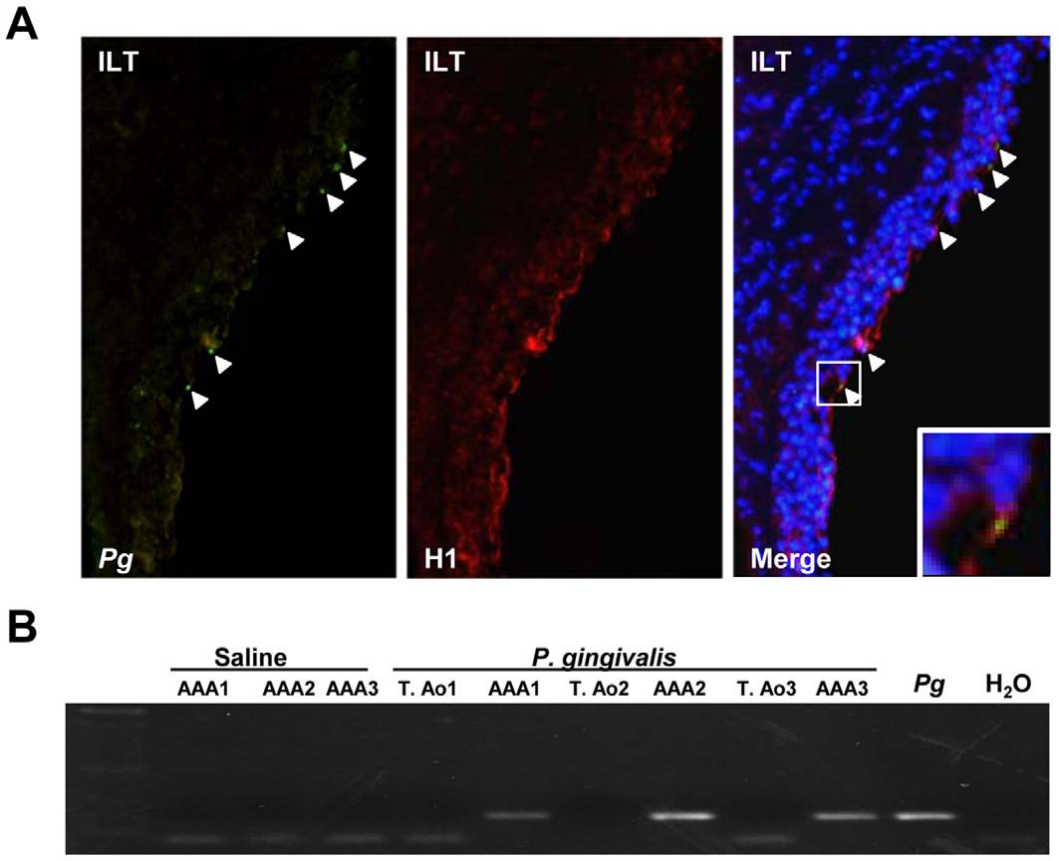
Thrombus colonization by *P. gingivalis*. (A) Immunostaining for *P. gingivalis* (*Pg*) in green and histone H1 (red). Merged images including DAPI staining (blue) show that *Pg* (white arrowheads) are trapped by extracellular nucleosomes (inset) at the interface of blood and intraluminal thrombus (ILT). (B) The presence of *Pg* was investigated by PCR using a set of primers targeting *Pg* 16SrRNA, in the AAA samples of saline-injected rats (AAA), in the thoracic (T. Ao) and in abdominal (AAA) aorta of *Pg*-infected rats. Amplification products were separated by electrophoresis in a 1% agarose gel.

In order to provide an experimental proof of concept that *P. gingivalis* may impact on aneurysm progression, we used the decellularized xenograft model of aneurysm in rats [29]. To mimic chronic bacteremia associated with periodontal disease, *P. gingivalis* was injected by the intravenous route once a week for 4 weeks (10^7^ CFU/rat) without producing significant modification of their general health status (no difference in body weight between control (346.3±6.8 g) and *P. gingivalis*-injected (338.7±1.9 g) rats, no signs of prostration and no macroscopically visible alterations of visceral organs: lung, liver and kidney at necropsy). Repeated *P. gingivalis* bacteremia induced a significant increase in the aneurysm size as compared to saline-injected rats (**Figure 7A, B**, median±IQR, *P. gingivalis* (n = 9): 8.12±2.68 mm *vs* saline (n = 11): 5.25±2.75 mm, p<0.03). Histological analysis showed that ILTs of rats infected by *P. gingivalis* were larger than those of non-infected rats and exhibited a significant enrichment in neutrophils (**Figure 8A**). As expected in this experimental model, mesenchymatous cell colonization associated with important fibrosis was observed in the ILT of non-infected rats, suggesting the beginning of the healing process (**Figure 8B**). In contrast, *P. gingivalis*-injected rats exhibited a large ILT containing neutrophils in the luminal part, mimicking what is observed in human pathology, without any sign of healing. We have also observed strong histone H1 immunostaining in the neutrophil-rich area associated with disorganized nuclear structure, suggesting the presence of NETs, similar to those observed in human ILT (**Figure 8C**). Double immunostaining for *P. gingivalis* and histone H1 showed the presence of *P. gingivalis* trapped by extracellular nucleosomes at the luminal pole of the ILT (**Figure 9A**). *P. gingivalis* colonization was shown to be specific of the ILT since neither the AAA of saline-injected rats nor the thoracic aorta of *P. gingivalis*-infected rats were positive for Pg 16S rRNA (PCR) (**Figure 9B**).

**Figure 10:**
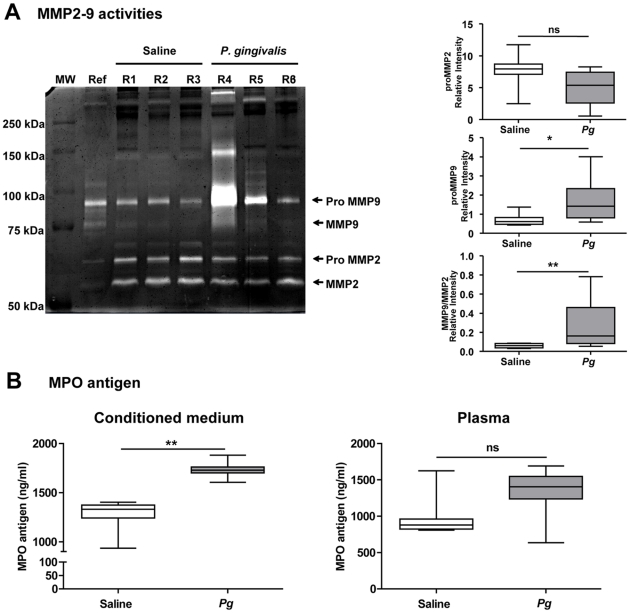
Increased MMP9 activity and MPO released by AAA samples of *P. gingivalis* (*Pg*) infected rats. (A) gelatin zymography analysis of saline- and *Pg*-injected rats (respectively rats R1,2,3 and R4,5,6). MW: molecular weight, Ref: reference containing pro- and active MMP-9. Graphs represent spatial density quantification of pro- and active MMP9 lysis areas (Image J software). (B) MPO concentration was determined by ELISA in conditioned medium and in plasma. *p<0.05, **p<0.01 (Mann-Whitney Analysis).

**Figure 11:**
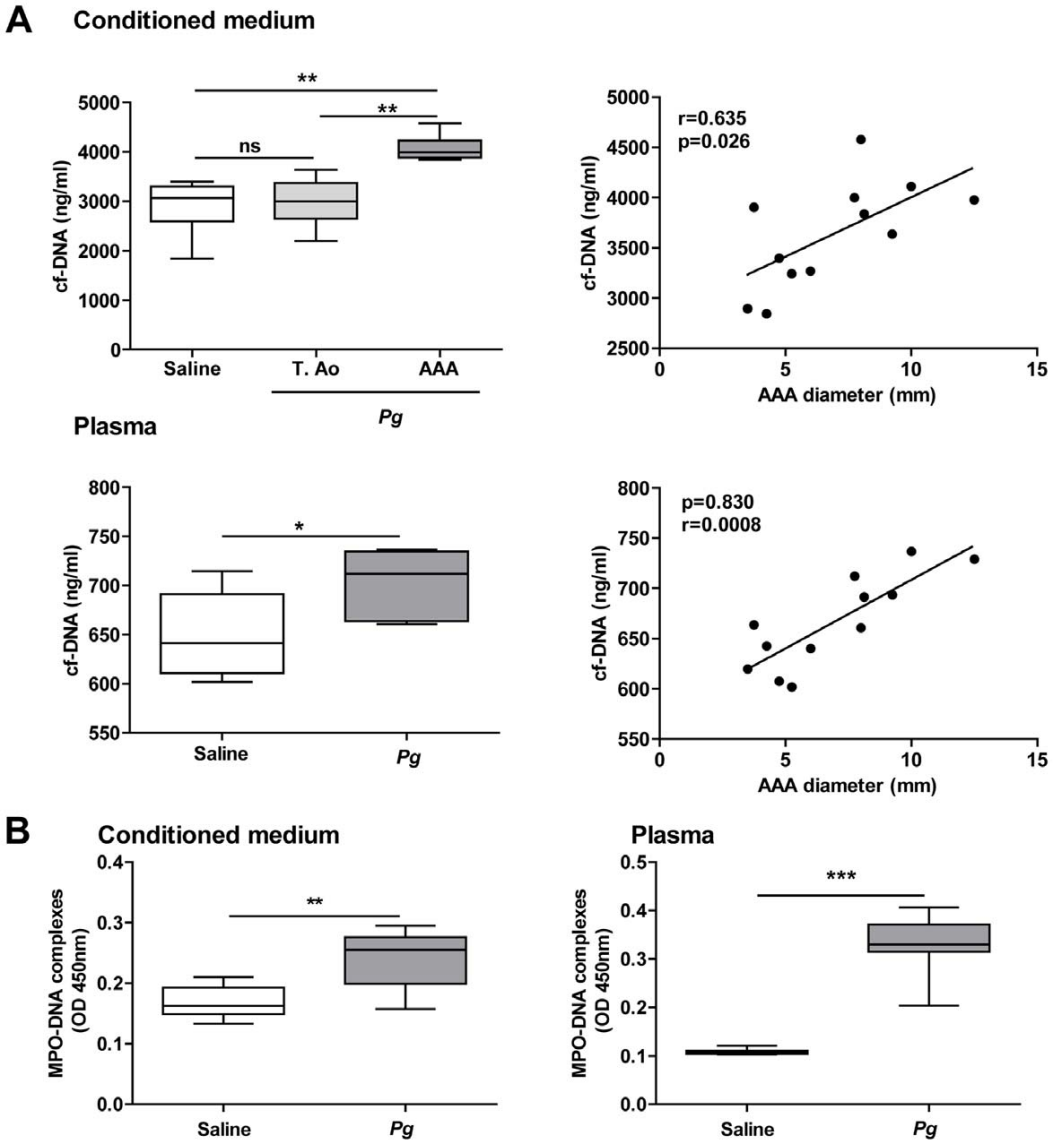
Cell-free DNA (Cf-DNA) and MPO-DNA complexes are increased in rats infected with *P. gingivalis* (*Pg*). (A) Concentration of cf-DNA released by the AAA segment (thrombus + wall) of saline- or *Pg*-infected rats or by the thoracic aorta (T. Ao) from *Pg*-infected rats. Cf-DNA was also quantified in plasma. (B) Quantification of MPO-DNA complexes in conditioned medium or plasma of saline-or *Pg*-injected rats. **p<0.01; ***p<0.0001 (Mann-Whitney analysis). The correlation between AAA diameter and Cf-DNA concentration was determined by the Least Squares method.

To further demonstrate the impact of neutrophil enrichment in the *P. gingivalis*-infected aneurysmal samples (wall+thrombus), MMP-2/MMP-9 activities (assessed by gelatin zymography), MPO and cf-DNA concentrations were measured in conditioned medium. MMP-9 activity was significantly increased in medium conditioned by AAA samples from *P. gingivalis*-infected rats compared to AAA samples obtained from non-infected rats (**Figure 10A**). In contrast, greater amounts of pro-MMP-2 were released by aneurysmal segments of non-infected rats relative to *P. gingivalis* -infected rats (p = 0.05), reflecting an ongoing healing process in non-infected rats, since pro-MMP-2 is preferentially secreted by mesenchymatous cells. The ratio MMP-9/MMP-2 was therefore significantly higher in conditioned medium from *P. gingivalis* -infected rats (0.163 vs 0.061 in saline-injected rats, p = 0.008). MPO concentration was strongly increased in AAA conditioned medium from *P. gingivalis*-infected rats as compared to non-infected rats (**Figure 10B**, p = 0.0003). In plasma, a trend towards increased MPO levels was observed in *P. gingivalis*-infected rats without reaching statistical significance (p = 0.065). The medium conditioned by the aneurysmal segment of the abdominal aorta contained more cf-DNA than the medium obtained by incubation of the adjacent aortic segment from the same rat (thoracic aorta, **Figure 11A**). Moreover, cf-DNA concentration was increased in both conditioned medium and plasma of rats injected by *P. gingivalis* compared to saline-injected rats (**Figure 11A** **)**. This suggests that cf-DNA is released by the AAA segment and not by the rest of the aorta potentially infected by *P. gingivalis*. More importantly, a strong positive correlation was observed between the concentrations of cf-DNA in both conditioned medium and plasma, and the AAA diameter (r = 0.635, p<0.03 and r = 0.83, p<0.001, respectively). Finally, NETs contributed, at least in part, to cf-DNA measured in plasma and conditioned medium since MPO/DNA complexes were detected in higher amounts in *P. gingivalis*-infected rat samples (**Figure 11B**).

## Discussion

Several lines of evidence led us to hypothesize that bacteria, and in particular periodontal pathogens, may participate in the development of AAA. Epidemiological data suggest an association between periodontal and cardiovascular diseases [39]. The nature of this association is however still a matter of debate; in particular, whether periodontal disease impacts directly on the pathogenesis of cardiovascular diseases or indirectly by increasing background inflammation is still not settled. Interestingly, atherothrombosis and periodontal disease share risk factors such as age, smoking and male gender [16], [40]. However, to date, no epidemiological study has been reported linking AAA development and periodontal disease. In the present study, we sought to establish a potential causal link between *P. gingivalis*, a highly prevalent periodontopathogen, and AAA progression.

### Neutrophil recruitment and activation in human AAA thrombus – Potential role of *P. gingivalis*

The persistence of neutrophils in the most luminal part of the ILT, interfacing with the bloodstream cannot be explained solely by their passive trapping during the process of fibrin formation. We have recently reported that conditioned medium from the luminal layer of ILT was chemoattractant for neutrophils in human AAA. However, blocking strategies interfering with the interleukin 8 pathway and RANTES only produced a 50% inhibition of neutrophil chemoattraction [10]. Since neutrophils constitute the first line of defense against bacteria and are strongly attracted by lipopolysaccharide (LPS) and bacterial peptides, we tested for the presence of endotoxin in AAA samples. We show that both the mural ILT and the residual AAA wall contained and released LPS that may account for neutrophil chemoattraction and activation. LPS from *P. gingivalis* was shown to stimulate neutrophils via LPS-binding protein in the serum of patients with periodontal disease [41]. *In vitro* studies have shown that neutrophil exposure to a variety of different microbial pathogens (*Staphylococcus Aureus, Escherichia Coli*), platelets, proinflammatory stimuli, hydrogen peroxide (H_2_O_2_), interleukin-8, or bacterial LPS, was able to trigger neutrophil extracellular trap (NET) formation. NETs were first described by Brinkmann *et al.* [35] as extracellular, highly decondensed chromatin structures released by activated neutrophils. In the present study, we have shown that stimulation of human neutrophils by *P. gingivalis* led to NET production, reflected by an increased cell-free DNA (cf-DNA) concentration in the culture supernatant and by histone exposure/modifications. The presence of NETs in the luminal part of the ILT, as well as in the adventitia of AAA samples, was shown by immunostaining of histone H1 and of the citrullinated form of histone H4. Massberg *et al.* recently suggested that NETs may promote fibrin formation *in vivo* and therefore limit pathogen dissemination [42]. Such a process could participate in the fibrin formation observed in the luminal part of the AAA thrombus. DNA and histones represent the major components of NETs and provide the backbone for the binding of neutrophil granule components (e.g. myeloperoxidase and elastase) that may play an antimicrobial role [36]. Histone 2A and 2B have also been shown to exhibit antimicrobial and endotoxin-neutralizing activities [43], reinforcing the bactericidal properties of NETs [44]. The presence of NETs in AAA samples was also associated with a release of cf-DNA, measurable in conditioned medium (ILT and adventitia). The release of cf-DNA by the adventitial layer may be due to local production of extracellular nucleosomes by activated neutrophils in response to bacterial stimulation coming from the vasa vasorum. Accordingly, cf-DNA levels were increased in plasma of AAA patients relative to control subjects. In addition, plasma cf-DNA levels were positively correlated with the abdominal aortic aneurysm diameter, suggesting that this marker may reflect the biological activity of the aneurysm and neutrophil activation in particular. Increased cf-DNA in plasma has been reported in pathologies other than AAA, involving neutrophil activation by bacteria, such as sepsis [38], [45] and appendicitis [36] or inflammatory processes such as small-vessel vasculitis [31]. Although some studies attribute cf-DNA to NETosis [38], caution must be exercised since different cell types may release circulating DNA upon necrosis, apoptosis or microparticle formation. We therefore measured MPO-DNA complexes in order to assess the amount of NETs in both conditioned medium and in plasma of patients versus controls. The same trend was observed as for cf-DNA: predominant release by the luminal part of the ILT and increased plasma levels in AAA patients relative to controls. In AAA, cf-DNA is a good marker of NET formation as attested by the positive correlation between cf-DNA levels and MPO/DNA complexes (r = 0.562, p = 0.0065).

### *P. gingivalis* in human AAA

The presence of bacterial DNA was investigated by PCR. DNA encoding for bacterial 16S ribosomal RNA was detected in 11/16 of the AAA samples tested whereas 7/16 were positive for detection of *P. gingivalis* DNA. These results are in line with a previous study [46], which tested the presence of 7 periodontopathic bacteria by PCR in AAA mural ILT and arterial wall and showed that more than 80% of AAA samples tested contained *P. gingivalis* DNA. The lower incidence in our study may be explained not only by different procedures for DNA extraction and primers used for detection of *P. gingivalis* DNA, but also by the higher prevalence of chronic periodontitis in the Japanese population compared with other developed countries[46]. In another recently published study by Nakano et al.[21], detection of *P. gingivalis* by PCR was reported in 8/76 aneurysm samples. However, no distinction was made between thoracic (TAA) and abdominal aortic aneurysms, which correspond to totally different pathologies. Interestingly, these authors distinguished between these two locations for assessment of the presence of *P. gingivalis* detection in dental plaque: 12/19 AAA were positive for oral *P. gingivalis* versus only 5/16 for TAA. More importantly, in our study, we have detected increased levels of anti- *P. gingivalis* antibodies released by AAA adventitia relative to those from normal aortic adventitia, probably associated with the presence of adventitial tertiary lymphoid organs in AAA [2]. In addition, serum titers of anti- *P. gingivalis* immunoglobulins were increased in AAA patients and strongly positively correlated with AAA thrombus volume (r = 0.731, p = 0.0001). This correlation is much stronger than those observed for other biological markers of AAA (i.e hemorphin 7/thrombus volume: r = 0.293 [28], MMP-9/AAA expansion: r = 0.33 [47], thioredoxin/AAA diameter: r = 0.5 [48]), and thus strengthens the potential link between *P. gingivalis* infection and AAA development. Interestingly, no correlation was observed between carotid intima-media thickness (IMT) and thrombus volume in our cohort of AAA patients (r = 0.25, p = 0.28). This suggests that *P. gingivalis* is specifically linked to AAA pathogenesis rather than being an additional risk factor increasing systemic inflammation associated with subclinical diffuse atherosclerosis (i.e increased IMT).

Other infectious pathogens such as *Chlamydia pneumoniae, Helicobacter pylori, Cytomegalovirus* or *Herpes simplex* virus have been suggested to be involved in the pathogenesis of AAA [49]. Whereas anti-*Chlamydia pneumoniae* antibodies were shown to be increased in plasma of AAA patients [50], most studies failed to demonstrate a link between infectious burden and AAA disease [49]. In addition, DNA corresponding to *C. pneumoniae* could not be detected in AAA samples [51], [52], [53]. Antibiotic strategies have been evaluated to thwart infectious agents and in particular *C. pneumoniae*. A study by Mosorin *et al.* [54] suggested that doxycycline may favorably alter the outcome of patients with AAA. Tetracyclines and particularly doxycycline, directly inhibit the activities of human matrix metalloproteinases (MMPs) in human AAA walls [55]. More recently, doxycycline was shown to reduce AAA neutrophil and cytotoxic T-cell content [56]. This antibiotic is known to reduce the severity and progression of periodontal disease in animal models and humans [57]. Doxycycline and derivatives have also the potential to inhibit the *P. gingivalis* proteinases such as gingipains [58], [59]. The beneficial effects of doxycycline on AAA progression could therefore also be attributed, at least in part, to its action against periodontal pathogens.

In contrast to lung or gastric bacteria, subgingival plaque pathogens can easily reach the bloodstream several times a day *via* chewing and toothbrushing, especially in patients with periodontal disease [60], [61]. Fimbriae and in particular fimbrillin A (FimA, the major fimbriae subunit) are involved in most adherence properties exhibited by *P. gingivalis*. Fim A was shown to promote adhesion to fibrin and fibrin/platelet matrices, which occur in ILT [62], [63]. Hemoglobin may also mediate *P. gingivalis* adhesion to the ILT and be used as a source of nutriment [25], [26]. However, since *P. gingivalis* are strict anaerobic bacteria, they are not likely to proliferate in an aerobic environment such as that observed in the ILT (PO_2_ = 100 mm Hg). Chronic bacteremia could therefore allow subclinical infection of different cardiovascular tissues including the ILT of AAA, principally composed of fibrin, platelets and hemoglobin.

### *P. gingivalis* in an experimental model of AAA in rats

In order to provide a proof of concept that *P. gingivalis* may be an actor of AAA progression, we have used an experimental model of AAA in rats [29]. This animal model is characterized by the formation of a thrombus about one week after grafting a decellularized guinea pig aorta in the abdominal position, associated with aortic dilation. In this model, like in all currently used AAA models, the mural ILT is rapidly colonized by mesenchymatous cells that initiate a fibrotic healing process. In contrast, after 4 weekly intravenous injections of *P. gingivalis*, the aortic diameter was not only significantly increased relative to saline-injected rats (p = 0.01), but the composition of the AAA was strikingly different. In *P. gingivalis*-injected rats, the mural ILT was persistent and exhibited a multilayered aspect, similar to what is observed in human AAA samples. The ILT was considerably enriched in neutrophils and all markers of their activation were increased in conditioned medium and in plasma of *P. gingivalis*- vs saline-injected rats. In the present study, we demonstrate that a periodontal pathogen enhances the development of AAA by maintaining the presence of a neutrophil-rich ILT, leading to a pathophysiological pattern similar to that observed in humans. It cannot be excluded that other bacteria or their products may have similar effects, and may participate in AAA pathogenesis, but our data highlight the major role of *P. gingivalis* in AAA development. Using a different model of experimental AAA in mice, Aoyama *et al.* [21] have reported that, in contrast to *P. gingivalis*, *A. actinomycetemcomitans* did not promote aortic dilation. However, these authors used a calcium-chloride model in mice, that consists in an external aggression of the aorta that does not lead to the formation of a thrombus, as often observed in humans.

### Strengths and limitations of the study

Our study provides a combination of clinical and experimental data that could link periodontal disease to AAA formation. However, albeit reaching statistical significance, the number of human samples analyzed is quite small. Additional epidemiological studies linking AAA and periodontal diseases would be necessary to support our findings. The model that we used is characterized by the formation of a thrombus about one week after xenografting. The healing process then usually takes place in the absence of additional aggression that would maintain the recruitment of neutrophils. In the present study, we report for the first time that chronic injection of *P. gingivalis* leads to the persistence of the ILT, similar to human pathophysiology. However, we did not test other periodontal pathogens that would be of interest, such as performed by Aoyama *et al.* [21]. Finally, we provide evidence that *P. gingivalis* DNA is present in AAA samples and that *P. gingivalis* material is sufficient to produce an adventitial immune response. However, the presence of living pathogens was not shown suggesting that *P. gingivalis* material, such as dead bacteria or LPS, may be sufficient to bind to the thrombus and promote its chronic renewal.

### Conclusion

In conclusion, the results of the present study indicate that *P. gingivalis* accelerates AAA progression via recruitment and activation of neutrophils, leading to production of NETs which are detectable in the plasma of AAA subjects. Because repeated subclinical episodes of bacteremia are systematically associated with periodontal diseases, *P. gingivalis* could be therefore a key actor in human AAA progression.

Taken together, our results demonstrate that a common pathogen may have a causal role in the pathogenesis of AAA. These findings bring significant new information to the field of AAA pathogenesis but should be strengthened by both epidemiological and observational studies in humans before one can envisage potential therapeutic strategies based on the treatment of periodontal disease to prevent AAA evolution towards rupture.
